# Macroscopic Quantum Tunneling of a Topological Ferromagnet

**DOI:** 10.1002/advs.202303165

**Published:** 2023-06-14

**Authors:** Kajetan M. Fijalkowski, Nan Liu, Pankaj Mandal, Steffen Schreyeck, Karl Brunner, Charles Gould, Laurens W. Molenkamp

**Affiliations:** ^1^ Faculty for Physics and Astronomy (EP3) Universität Würzburg Am Hubland D‐97074 Würzburg Germany; ^2^ Institute for Topological Insulators Am Hubland D‐97074 Würzburg Germany

**Keywords:** macroscopic quantum tunneling of magnetization, magnetism, quantum anomalous hall effect, topological insulators

## Abstract

The recent advent of topological states of matter spawned many significant discoveries. The quantum anomalous Hall (QAH) effect is a prime example due to its potential for applications in quantum metrology, as well as its influence on fundamental research into the underlying topological and magnetic states and into axion electrodynamics. Here, electronic transport studies on a (V,Bi,Sb)_2_Te_3_ ferromagnetic topological insulator nanostructure in the QAH regime are presented. This allows access to the dynamics of an individual ferromagnetic domain. The domain size is estimated to be in the 50–100 nm range. Telegraph noise resulting from the magnetization fluctuations of this domain is observed in the Hall signal. Careful analysis of the influence of temperature and external magnetic field on the domain switching statistics provides evidence for quantum tunneling (QT) of magnetization in a macrospin state. This ferromagnetic macrospin is not only the largest magnetic object in which QT is observed, but also the first observation of the effect in a topological state of matter.

## Graphical abstract

The profound question of how the laws of quantum mechanics merge into those governing classical physics at macroscopic sizes remains largely unexplored. Here quantum tunneling of the magnetization, a phenomenon quantum by nature, is studied in a magnetic object that is, without any doubt, macroscopic.

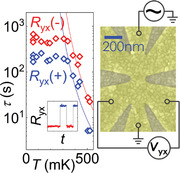

## 1 Introduction

Since the birth of quantum mechanics in the early twentieth century, physicists have studied how the laws of quantum mechanics merge into those governing classical mechanics at macroscopic sizes. One of the more prominent consequences of quantum mechanics is the phenomenon of quantum tunneling (QT) between two separate eigenstates, an effect that has no direct analog in classical physics. This poses an intriguing question regarding the possibility of QT effects in systems that can be regarded as macroscopic. In the past four decades, experimental techniques have started to reach the level of sophistication needed to explore this issue. The topic has since been explored in studies of macroscopic QT between different current states in Josephson junctions,^[^ ^1^, ^2^, ^3^ ^]^ macroscopic QT of the magnetization, ^[^ ^4^, ^5^, ^6^, ^7^, ^8^, ^9^, ^10^, ^11^ ^]^ and macroscopic QT of magnetic domain walls^[^ ^12^, ^13^ ^]^ in magnetic structures. The same period also saw the rise of topology in condensed matter,^[^ ^14^, ^15^ ^]^ as topological insulators (TIs),^[^ ^15^, ^16^ ^]^ the first experimentally demonstrated topological state of matter,^[^ ^17^ ^]^ opened a path toward new quantum phenomena such as the quantum spin Hall effect^[^ ^16^, ^17^, ^18^ ^]^ in non‐magnetic systems, and the quantum anomalous Hall (QAH) effect^[^ ^19^, ^20^, ^21^ ^]^ in ferromagnetic TIs. The latter is especially interesting due to its potential for applications in quantum metrology,^[^ ^22^, ^23^ ^]^ as well as its influence on fundamental research into the underlying topological and magnetic states^[^ ^24^, ^25^, ^26^, ^27^, ^28^, ^29^ ^]^ and into axion electrodynamics.^[^ ^20^, ^30^, ^31^, ^32^ ^]^ In this article, we investigate the QAH state in a nanostructure with a mesa constriction as small as 160 nm, fabricated from a V‐doped (Bi,Sb)_2_Te_3_ magnetic TI layer. This size regime allows us to access and carefully investigate the dynamics of an individual magnetic domain in the material. The influence of temperature and magnetic field on the domain switching statistics shows that in the low temperature limit, the switching is governed by macroscopic QT of magnetization.

## 2 Quantum Anomalous Hall Nanostructure Device

**Figure 1:**
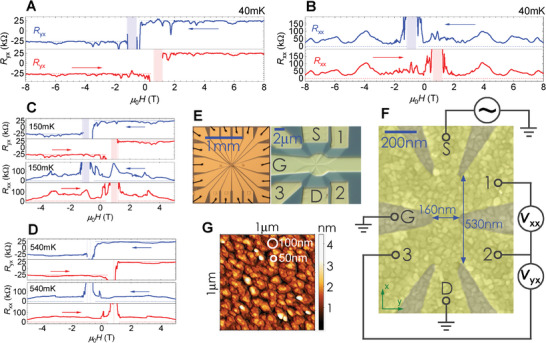
Quantum anomalous Hall (QAH) measurements on a (V,Bi,Sb)_2_Te_3_ nanostructure. A) Hall resistance (*R*_yx_), and B) longitudinal resistance (*R*_xx_), collected at temperature 40 mK as a function of magnetic field. C,D) Same, for higher sample temperatures of 150 mK (C), and 540 mK (D). The arrows indicate the magnetic field sweep direction. The shaded regions represent a regime around the global magnetization reversal where the two‐terminal resistance of the sample exceeds 4 MΩ, and the four‐terminal measurement scheme becomes invalid. The horizontal dashed lines indicate the resistance values expected from a perfect QAH effect. E) Optical microscope images of the actual device used in the experiment, left: a low magnification image of the entire sample, right: an image of the nanostructure with the contact labelling (S: source, D: drain, G: gate, and 1–3: voltage probes). F) High magnification false color SEM image of a reference device together with a circuit diagram schematic. The granularity visible on the picture is the surface of the gate metal. G) Atomic force microscope (AFM) image of a 1 µm by 1 µm scan of the surface topography of a reference uncapped (V,Bi,Sb)_2_Te_3_ layer, exemplifying the crystal twinning structure in the film. The white circles, positioned arbitrarily in the figure, are merely visual aids indicating the estimated range of sizes (50–100 nm) for the magnetic domain.

Our magnetic TI device is patterned from an 8.2 nm thick V_0.1_(Bi_0.2_Sb_0.8_)_1.9_Te_3_ layer grown by molecular beam epitaxy (MBE) on a Si(111) substrate.^[^ ^33^ ^]^ It is capped in‐situ with a 10 nm thick Te layer, and the growth conditions are optimized for perfect anomalous Hall resistance quantization, as observed previously in our macroscopic‐size devices.^[^ ^22^, ^25^ ^]^ A high magnification scanning electron microscope (SEM) image of a reference device is shown in **Figure** **1**F. The six‐terminal nanostructure has a width of about 160 nm and a length of about 530 nm (see blue arrows in Figure 1F for how these dimensions are defined), and is patterned using a combination of electron beam lithography (for the mesa etched using Ar milling and AuGe ohmic contacts) and optical lithography (for the AlOx/HfOx/Au gate plus dielectric layer stack). More details about the lithography can be found in the supporting information. Indeed, the investigations of sub‐micron size QAH devices have recently attracted attention in the community, as down‐scaling provides access to some new transport properties.^[^ ^34^, ^35^ ^]^ While the sample is fitted with a top gate, for simplicity of the analysis the gate electrode is grounded for all experiments discussed in this article (some measurements with an applied gate voltage can be found in Figure S1, Supporting Information ). The influence of gate voltage on the physics will require further research and thus be the subject of future work.

## 3 Results

Figure 1 presents basic magneto‐transport data. The magnetic field direction is always perpendicular to the plane of the sample. A low frequency AC voltage excitation with an amplitude of about 100 µV is applied between the source contact “S” and the grounded drain contact “D”, while contacts one through three are used to probe the transverse (Hall) *V*_yx_ and longitudinal *V*_xx_ voltages (a simplified circuit schematic is given in Figure 1F). The current *I* is of the order of 0.2 nA, and is obtained by measuring the voltage drop over a calibrated reference resistor (about 50 kΩ) connected in series with the sample. A two‐terminal resistance measurement corresponding to the data in Figure 1A,B can be found in the Figure S6 (Supporting Information). The Hall resistance (*R*_yx_=*V*_yx_/*I*) of close to ±*h*/*e* ^2^ visible in Figure 1A shows that the device is in the QAH regime. The small and fluctuating deviations from the perfectly quantized value are primarily a result of longitudinal voltage admixing. Indeed when the symmetric (with respect to the hysteretic *B* field) component is subtracted from the measured *R*_yx_ signal, the accuracy of the quantization improves significantly (see Figure S5, Supporting Information ). The accuracy of quantization at zero magnetic field is comparable to that recently reported in a nanostructure of similar size, but based on a Cr‐doped (Bi,Sb)_2_Te_3_ material,^[^ ^35^ ^]^ and in the case of our nanostructure is approx. ρ_yx_=1.01 *h*/*e* ^2^ (after removing the symmetric component), with a longitudinal signal of about ρ_xx_=0.45 *h*/*e* ^2^. This finite ρ_xx_ implies an apparent deviation from quantization larger than 0.01 *h*/*e* ^2^. Since the larger Hall bars fabricated from this material exhibit robust QAH quantization with vanishing longitudinal resistance^[^ ^22^, ^25^ ^]^ (also see Figure S7, Supporting Information ), the finite longitudinal resistance (*R*_xx_=*V*_xx_/*I*) visible in Figure 1B may indicate the influence of sample size effects. For example, the edges of the device are close enough to each other to either directly couple due to the edge state wavefunction overlap,^[^ ^36^ ^]^ or more likely due to a path of topological channels extending into the bulk by meandering through the magnetic domain structure. This of course also contributes to deviations of *R*_yx_ from an otherwise perfectly quantized value. In the end, both measured *R*_xx_ and *R*_yx_ signals are likely a consequence of a complicated Landauer‐Büttiker network of 1D channels formed around the magnetic domains profile within the material. Another possible explanation for finite *R*_xx_ and nearly quantized *R*_yx_ is the emergence of a so‐called anomalous Hall insulator state,^[^ ^37^ ^]^ induced by magnetic disorder in the material. This however can likely be excluded in our sample, as larger devices patterned from the same MBE grown layer show a vanishing *R*_xx_ and quantized *R*_yx_ signals, reminiscent of a robust QAH insulator (see Figure S7, Supporting Information ). Interestingly the value of the longitudinal resistance close to the coercive field in our nanostructure is significantly larger than that of large devices based on the same layer thickness.^[^ ^32^ ^]^ An explanation of this difference will require further investigation and may provide some clues to the nature of the percolating channels formed when the material breaks into domains during the global magnetization reversal. An important point to note for the upcoming analysis is the clear difference in transport behavior observed between the 150 mK (Figure 1C) and the 40 mK (Figure 1A,B) measurements, implying that the lowest temperature experienced by the carriers in the sample is well below 150 mK in this experiment.

**Figure 2:**
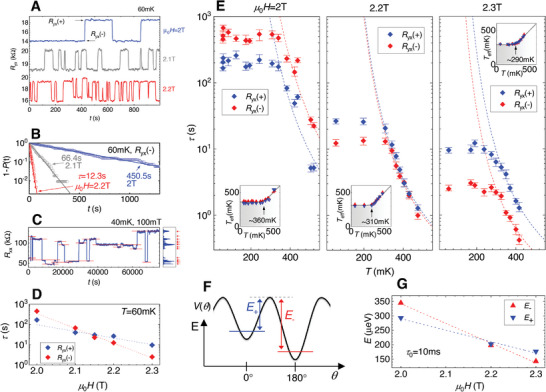
Macroscopic quantum tunneling of magnetization. A) Typical time dependent measurement of *R*_yx_ at various magnetic field and a temperature 60 mK, showing the telegraph noise in the signal. The “+” and “‐” label the larger and smaller *R*_yx_ states, respectively. B) Probability of the state not being switched after a given time in the *R*_yx_(‐) state, along with the fitting to an exponential decay function, for the three magnetic field values from (A). C) An *R*_xx_ measurement collected at a field of 100 mT, where we observe the largest number of discrete states (13) at base temperature. The horizontal red lines and the “bin counter” next to the figure help identify the number of distinct states. D) Evolution of the lifetime τ with magnetic field at 60 mK. E) Evolution of τ with temperature for three magnetic field values. The error bars represent a conservative range of values of τ that can describe the exponential decay. The insets show the effective temperature (*T*_eff_), saturating in the low temperature regime (see text for details). F) A simplified schematic of the anisotropic potential landscape for the magnetization, with energies defined: *E*_+_ and *E*_−_ are the thermal activation energy for each state. An angle θ of 0 or 180 degrees is along the easy axis of the ferromagnet (which is normal to plane of the sample). G) Evolution of the activation energies with the external magnetic field. The values correspond to the colored dashed lines in E) following τ=τ_0_exp[*E*_+/ −_/(*k*_B_ *T*)], with τ_0_=10 ms for all six curves.

**Figure** **2**A highlights the key observation of this study. Here, the Hall resistance of the sample is recorded as a function of time, at three values of magnetic field (µ_0_ *H*=2 T, 2.1 T, and 2.2 T), and at a temperature 60 mK. *R*_yx_ is manifestly not constant with time. This is in stark contrast to measurements in macroscopic (hundreds of microns wide) QAH devices, where no instabilities are observed (except for during magnetic reversal at the coercive field of the ferromagnet).^[^ ^26^ ^]^ The resistance instability has a two‐level telegraph noise signature,^[^ ^6^, ^38^, ^39^ ^]^ suggestive of an individual magnetic domain switching between two magnetization states. Indeed, the telegraph noise statistics vary systematically with the external magnetic field strength and temperature, and also follow the behavior expected from an individual tunneling magnetic domain. This excludes alternative mechanisms (such as charge trapping) as the source of the telegraph noise. For each of the measurements, the sample was magnetically prepared by first applying a field of 7.5 T, and ramping it back down to the specified value in the same direction, i.e., without crossing zero. We thus emphasize that the switching occurs spontaneously despite the magnetic field still being applied in the same direction as the magnetization of the majority of the domains in the sample, and thus being far away from the coercive field that would trigger a global magnetization reversal. Interestingly, no such domain instabilities were previously observed in Cr‐doped (Bi,Sb)_2_Te_3_ material using ultra low temperature scanning SQUID and MFM magnetometric methods.^[^ ^24^, ^40^ ^]^ We speculate that due to the significant differences in magnetic anisotropy between Cr‐ and V‐doped materials, the frequency range associated with the domain dynamics that drive the telegraph noise could differ significantly for both materials (if the dynamics are significantly faster in a weaker anisotropy Cr‐doped material, the magnetization fluctuations may be much more challenging to investigate). Another possible factor is the measurement temperature, which in [24] is 250 mK and in [40] is 500 mK, which in both cases may be too large to observe telegraph noise.

To properly analyze this telegraph noise, each measurement is allowed to run sufficiently long to record about 200 (or more) switching events. The duration of the plateaus between the switching events is then extracted, distinguishing between jumps from the “+” (larger *R*_yx_) or from the “‐” (smaller *R*_yx_) state. From this data, we can determine the probability *P*(*t*) of the state having switched to the other state after having been in a given state for a time *t*. The probability of the state not having switched by time *t* is then obviously 1‐*P*(*t*), which for a random process with a mean lifetime τ follows an exponential decay exp(‐*t*/τ).^[^ ^10^, ^38^ ^]^ An example of this for the *R*_yx_(‐) state is plotted as the symbols in Figure 2B, along with an exponential decay fit, with the decay constant τ indicated in the figure giving the lifetime of the state under the given conditions.

When time dependent measurements are performed at magnetic fields far away from the range focused on in Figure 2 (which ranges from 2 T to 2.3 T), we observe magnetic switching between significantly more than two states, indicating that multiple magnetic domains are active. An example of this is shown in Figure 2C for 100 mT, and similar behavior is also observed when a gate voltage is applied; see Figure S1 (Supporting Information). It is thus incidental that in the magnetic field range close to 2 T, only a single magnetic domain contributes to the instability. Given the high sensitivity of lifetime τ to the external magnetic field (as presented in Figure 2D) it cannot be ruled out that other domains are also active, but that their contributions are either much too slow or much too fast, relative to the measurement time and data acquisition speed, to be perceived. We also note that large time instabilities are observed even at fields as large as 8 T, which implies that this magnitude of magnetic field is still not sufficient to fully saturate the magnetization of all elements in this material, despite the coercive field being only about 1 T. This is in line with our previous observations in larger devices^[^ ^25^ ^]^ (10 microns and more), where the nature of resistance fluctuations observed near the coercive field was strongly dependent on the magnitude of the previously applied (preparing) magnetic field in the opposite direction, with changes observed even for fields as large as 16 T.

For a rough estimate of the size of the domain that is responsible for the switching analyzed in Figure 2, we first turn to the data in Figure 2C. The largest amount of distinct states observed in our device in any base temperature measurement occurs for a field of 100 mT, where 13 levels are seen (Figure 2C). More measurements at various magnetic field values at base temperature can be found in Figure S2 (Supporting Information), all showing less than 13 states. These 13 states imply at least four active domains (which could produce a maximum of 2^4^=16 independent states). Given that the measured Hall signal never changes sign, one can assume that these four magnetic domains correspond to less than half of the device area. If the device area is taken as a 160 nm by 530 nm rectangle (blue arrows in Figure 1F), this results in an upper bound on the magnetic domain size of order 115 nm in diameter. While this number provides only an upper bound, the real domain size cannot be significantly smaller than 115 nm. The number of available states increases exponentially with reduced domain size, such that it would quickly lead to significantly more than the 13 observed levels in Figure 2C. Moreover, the amplitude of the resistance change (in Figure 2A,C) is of the order of a few tens of percent during the switching events, which implies that the switching domains must represent a sizable fraction of the device area. Furthermore, it is plausible that the crystal domain structure within the film (visible in the AFM image of a reference uncapped film; Figure 1G), which breaks the translational symmetry at the crystal domain boundaries, determines the magnetic domain landscape. These crystal rotational twin domains are in the 50–100 nm size range. Finally, previous magneto‐metric imaging experiments by other groups on similar layers revealed the magnetic domain sizes in the 50–200 nm range.^[^ ^24^, ^40^ ^]^ Considering all the above, it is reasonable to infer the magnetic domain size to be in the 50–100 nm range.

It is natural to assume that the state with a larger *R*_yx_ (labeled with the “+” index in Figure 2A) represents a magnetic domain aligned in the same direction as the background magnetization, i.e., ferromagnetically coupled to it's surroundings (and thus adding to the total Hall signal), while the lower *R*_yx_ state (labeled “‐”) has the domain antiferromagnetically coupled to the surroundings. This interpretation is supported by the evolution of the lifetime τ of each state as a function of the external magnetic field, as analyzed in Figure 2D. At a magnetic field of 2 T, the “‐” state has the larger lifetime, and is therefore the energetically more stable state. As the magnetic field is increased, the lifetimes of the two states cross, and above about 2.15 T the “+” state becomes more stable, consistent with the external magnetic field favoring the ferromagnetic alignment of the domain, over the antiferromagnetic alignment.

Despite the fact that both states cross, each lifetime is monotonically decreasing with the external magnetic field strength. The overall trend for each lifetime in Figure 2D is a complicated product of, at least, the direct interaction of the domain with the external field, the magnetic landscape in the vicinity of the domain (i.e., its magnetic coupling to neighboring domains, a mechanism of which remains an open question in this material system), and details of the domain size and shape. Without full microscopic information with regard to a specific domain and its surroundings, it is impossible to quantitatively predict the exact external magnetic field dependence of this signal. Despite that, some qualitative behavior can be expected. If the local domain configuration is such that, in the ground state without any external magnetic field, the domain in question happens to be aligned opposite to the majority of the domains (i.e., the “‐” state is energetically more stable), then as the magnetic field is increased, the energy barrier between its two magnetization states will in general decrease (as the external magnetic field is applied in the direction opposing the ground state magnetization direction of the domain). This explains the trend in Figure 2D and the activation energies in Figure 2G. Indeed, it is quite likely that our experiment, by its nature, selects out just such a domain, as a domain that already at zero magnetic field has a clear ground state with parallel magnetization to the majority, is likely to be only further stabilized by the magnetic field, and will never exhibit any observable dynamics. For the same reasons it is unsurprising that the external magnetic field strength scales associated with the individual domain dynamics (about 2.1 T in Figure 2D, and 3.2 T in Figure S8A, Supporting Information , for two distinct magnetic domains from different samples) can in general differ from global material parameters, such as the coercive field (about 1 T), which result from an ensemble behavior of the domains, and do not reflect the properties of an individual one.

In Figure 2E we turn to the temperature dependence of τ for each state, and for three magnetic fields. In the high temperature regime the lifetime follows an Arrhenius like thermal activation τ=τ_0_exp[*E*_+/ −_/(*k*_B_ *T*)] (colored dashed lines), where *E*_+/ −_ is the activation energy for each state, and *k*_*B*_ the Boltzmann constant. This is consistent with the behavior expected from a magnetic thermal agitation.^[^ ^6^, ^9^, ^10^, ^13^, ^38^, ^39^ ^]^ We find a good match for all six curves (“+” and “‐” states for each magnetic field) with τ_0_=10 ms. This is the value used for the dashed lines in the figure, but values ranging from 1 to 20 ms also describe the data reasonably well (see Figure S4, Supporting Information). The exact value of τ_0_ quantitatively affects the obtained energies (*E*_+_ and *E*_−_), but the roughly linear dependence of these energies on magnetic field, shown in Figure 2G, is robust to the uncertainty in τ_0_.

Values of τ_0_ reported in the literature span a wide range from nanoseconds to milliseconds,^[^ ^6^, ^9^, ^10^, ^13^, ^38^, ^39^ ^]^ putting our values at the upper end of that range. This wide spread of value reflects the fact that there is no clear physical interpretation of the prefactor τ_0_. It is best viewed as a phenomenological parameter that aims to express the effects of size and shape anisotropy on a magnetic object and its interactions with its neighbors and its environment, as a single number.

Much more interesting and fundamental, however is the behavior in the low temperature regime. Here, the Arrhenius thermal activation picture breaks down, as the lifetime saturates, forming a large plateau in the temperature dependence of τ. The dynamics are clearly no longer governed by thermal agitation, where the magnetization during the reversal process follows a classical trajectory over the anisotropy induced energy barrier. Rather, the quantum limit has been reached, and the switching rate depends solely on the tunneling probability through the potential barrier,^[^ ^4^ ^]^ a trajectory forbidden in classical physics. Indeed, such a low temperature saturation of magnetic dynamics, followed by a high temperature thermally activated regime, was previously attributed to QT in a number of magnetic systems.^[^ ^6^, ^9^, ^10^, ^13^, ^39^ ^]^ In order to better identify the transition temperature, the data in Figure 2E can be used to generate it's insets. Here the *x* axis gives the temperature for each measurement, whereas the *y* axis gives an effective temperature (*T*_eff_) which would be associated with the given lifetime within the thermal activation model (for a given measured lifetime τ_data_, the effective temperature *T*_eff_ is obtained by solving the equation τ_data_=τ_0_exp[*E*_+/ −_/(*k*_B_ *T*_eff_)] for *T*_eff_). Plotted in this way, a clear inflection between the two regimes is visible, as marked by the arrows in the insets.

As visible in Figure 2G, the thermal activation energies (and therefore the potential barrier height for each state), as extracted from the high temperature regime, decrease significantly as magnetic field is increased (from about 300 to 150 µeV when the magnetic field is changed from 2 to 2.3 T). A quantum mechanical wavefunction penetrating into a potential barrier, to a good approximation, decays exponentially, with a decay constant strongly dependent on the barrier height. Therefore, the tunneling probability is expected to significantly increase when the barrier height is moderately reduced. This explains the roughly exponential decrease of τ for each state with increasing magnetic field that is shown in Figure 2D for 60 mK, a temperature deep in the QT regime. In addition we observe a decrease in the onset point of saturation of *T*_eff_ with increased magnetic field. The insets in Figure 2E show that the *T*_eff_ plateau develops at a temperature of about 360 mK at magnetic field of 2 T, at about 310 mK at 2.2 T , and at about 290 mK at 2.3 T. This is likely a result of a complicated balance between the tunneling rate and the thermal agitation rate, as both can in principle evolve differently with the potential barrier height. Nevertheless, the fact that the observed transition temperature reacts to the magnetic field, further demonstrates that the observed low temperature saturation of dynamics is governed by the magnetic properties of the device.

## 4 Discussion

Over the past 25 years or so, investigations into QT of magnetization have primarily focused on tunneling in large ensembles of magnetic molecules.^[^ ^7^, ^8^, ^9^, ^41^ ^]^ We stress that while QT in such individual molecules is frequently referred to as “macroscopic”, it is fundamentally a collective response of an ensemble of identical microscopic objects. Each molecule has a size of order 1 nm, and a spin resulting from only the handful of magnetic atoms within the molecule. This leaves open room for debate as to how truly “macrosopic” such tunneling is. Only a few works have reported on QT of magnetization in somewhat larger magnetic structures. This includes molecules of antiferromagnetic ferritin where the magnetic core, which is comprised of up to 4500 Fe atoms, has a diameter of only about 7 nm,^[^ ^11^ ^]^ as well as lithographically patterned SrRuO_3_ nanostructures where the tunneling volume was found to be close to 100 nm^3^^[^ ^42^ ^]^ (i.e., a few nanometers in diameter). The only reported evidence for QT of magnetization observed in an individual magnetic nanocrystal (ferrimagnetic barrium ferrite) can be found in Ref. [10]. There, though not as clearly pronounced as in our results, the Authors observed a clear onset of saturation of the magnetic dynamics at low temperature, corresponding to macroscopic QT. The system there is a disk about 10–20 nm in diameter.

In contrast, we observe QT of a ferromagnetic macrospin originating from a dilute ferromagnetic domain spread over a clearly macroscopic distance of 50–100 nm. While this is a rough estimate, the conservative end of the scale corresponds to a magnetic domain size of 50 nm in diameter, representing a volume of some 16 000 nm^3^ and containing 10 000 magnetic atoms. This volume is significantly larger than any previously studied macrospin. Our results thus not only provide the most robust evidence of QT of a truly macroscopic magnetic state reported to date, but also provide first realization of this effect in a topological state of matter. This in turn opens a path toward investigating possible intriguing connections between macroscopic quantum phenomena and topology.

## 5 Conclusion

We have fabricated a (V,Bi,Sb)_2_Te_3_ TI nanostructure in the QAH regime. The size of the device is small enough that the dynamics of an individual ferromagnetic domain can be resolved, with a collective magnetic moment spread over a macroscopic distance of 50–100 nm. Careful analysis of the influence of magnetic field and temperature on the telegraph noise originating from the domain magnetization fluctuations, provides strong evidence of QT of a truly macroscopic magnetization state, and that in a TI.

## Author Contributions

K. M. F. designed and patterned the measured devices, performed the transport experiments, and the telegraph noise analysis. N. L. grew the sample. P. M. contributed to nanolithography development and fabricated the reference device. S. S. contributed to growth optimization. K. B., C. G., and L. W. M. supervised the work. All authors contributed to the analysis and interpretation of the results, and the writing of the manuscript.

## Conflict of Interest

The authors declare no conflict of interest.

## Supporting information

Supporting Information

## Data Availability

The data that support the findings of this study are available from the corresponding author upon reasonable request.
