# Supplementary material for: Macroscopic Quantum Tunneling of a Topological Ferromagnet
Source: Adv Sci (Weinh). 2023 Jun 14;10(22):2303165. doi: 10.1002/advs.202303165 (PMC10401085; doi:10.1002/advs.202303165)
Supplement: Supplementary file 1 — Supporting Information [file ADVS-10-2303165-s001.pdf]

ADVANCED SCIENCE
Open Access

Supporting Information

for *Adv. Sci.*, DOI 10.1002/advs.202303165

Macroscopic Quantum Tunneling of a Topological Ferromagnet

Kajetan M. Fijalkowski, Nan Liu, Pankaj Mandal, Steffen Schreyeck, Karl Brunner, Charles Gould* and Laurens W. Molenkamp*

Supporting information

Macroscopic Quantum Tunneling of a Topological Ferromagnet

Kajetan M. Fijalkowski, Nan Liu, Pankaj Mandal, Steffen Schreyeck, Karl Brunner, Charles Gould, and Laurens W. Molenkamp

Table of contents:

1. Magnetic topological insulator layer growth.
2. Lithography.
3. Transport measurements.
4. Time dependent measurements collected at various applied gate voltages for a 160 nm wide device.
5. Time dependent measurements collected at various applied magnetic fields for a 160 nm wide device.
6. Comparison between an estimated magnetic domain size in a 160 nm wide device and the surface topography of the film.
7. Thermal activation analysis for various values of parameter τ_0 in a 160 nm wide device.
8. Anti-symmetrization procedure for magnetic field sweeps of Hall resistance in a 160 nm wide device.
9. Two-terminal resistance of the 160 nm wide device at 40 mK.
10. Summary of the results from a series of Hall bar devices of various sizes, patterned from the same MBE layer as the 160 nm device.
11. Macroscopic quantum tunneling of magnetization in a 240 nm wide device.

1. Magnetic topological insulator layer growth.

The investigated magnetic topological insulator $V_{0.1}(Bi_{0.2}Sb_{0.8})_{1.9}Te_3$ layer is grown using molecular beam epitaxy (MBE) on an H-passivated, intrinsic, Si(111) substrate. The layer is capped in-situ with Te, as a protection from ambient conditions and during lithography. The magnetic topological insulator layer thickness, along with the thickness of the Te cap, are determined using a combination of X-ray reflectivity (XRR) measurements of capped, and reference uncapped films. The Te cap is 10 nm thick, and the $V_{0.1}(Bi_{0.2}Sb_{0.8})_{1.9}Te_3$ layer is 8.2 nm thick. The substrate temperature is kept at 190˚C during the growth. The Bi/Sb ratio of ¼ and V doping of 2% (of all atoms) is determined by X-ray diffraction (XRD) and energy-dispersive X-ray spectroscopy (EDS) measurements of the thin film, as well as bulk calibration samples (~50 nm thick in order to obtain sufficient characteristic X-ray signal from vanadium atoms).

2. Lithography.

The four smallest Hall bars (160 nm, 240 nm, 1 µm, and 2 µm), which includes the two nano Hall bars in which the quantum tunneling is observed, are patterned using a combination of optical and electron beam lithography.

- In the first lithographic step, the global alignment markers and the local write field alignment markers are exposed using electron beam. This is followed by resist (PMMA) development, Ti/Au 3 nm/100 nm layer stack evaporation, and lift-off in acetone.

- In the second step the mesa structure is patterned. Here the electron beam exposure is followed by a development, 20 nm thick Ti layer evaporation and a lift-off. Ti layer acts as a hard mask for the etching. Sample is then dry etched using Ar ion beam etching (IBE). Afterwards the Ti mask is removed using buffered oxide etch.
- In the third step, the electrical contacts are patterned. The electron beam exposure is followed by development, Ar milling to etch through the Te cap, and evaporation of a AuGe/Ti/Au 50 nm/5 nm/50 nm layer stack to form a good electrical contact to the Hall bar. This is followed by lift-off in acetone.
- In the fourth step, the gate dielectric and electrode are defined. First the sample is placed in the atomic layer deposition (ALD) chamber, where a layer stack of AlOx/HfOx 13 nm/ 2nm is deposited at a temperature of 90˚C (on the entire surface of the sample). This is followed by defining the gate electrode layer stack of Ti/Au 5 nm/100 nm using standard optical lithography methods.
- In the fifth and final lithographic step, the big metal pads (for wedge bonding) are patterned: First, the optical resist structure is developed, then the sample is submerged in highly diluted hydrofluoric acid (HF) (1:200 with water) to etch through the dielectric (from the previous step) on the big contact pads. This is followed by evaporation of a Ti/Au 5 nm/ 150 nm layer stack to ensure good electrical contact with all previously patterned metal pads on the sample. This is then followed by an acetone lift-off.

The two largest samples (8 μm and 200 μm wide Hall bars, used to acquire the data of Fig. S7) are patterned using standard optical lithography methods.

3. Transport measurements.

All transport measurements are performed in a dilution refrigerator with a base temperature of about 40-60 mK, equipped with a mixing chamber heater allowing stabilization at higher temperatures, and a superconducting solenoid magnet producing a perpendicular to plane external magnetic field (with respect to the sample plane).

Electrical measurements are performed using conventional high precision and low frequency lock-in techniques. Signals from the sample (longitudinal or Hall voltage) are fed into ultra-high input impedance operational amplifier (input impedance ~ 1 TΩ), and the output of this amplifier (output impedance ~ 50 Ω) is fed into the lock-in. This avoids any issues with the relatively low input impedance of the lock-ins (~ 10 MΩ) that would otherwise introduce artifacts on the measurements since the resistance of the sample reaches a non-negligible fraction of the lock-in input impedance (Fig. S6 shows a two-terminal resistance measurement of the nanostructure). The measurement frequency is usually below ~20 Hz, with exception of the telegraph noise measurements at higher temperatures (where the dynamics significantly speed up) and a frequency increased to ~200 Hz is needed. An AC voltage with an amplitude of about 100 μV is applied to the sample (a voltage biased scheme), and side contacts of the Hall bar are used to probe the Hall and longitudinal voltage signals. The current is measured using a calibrated series reference resistor (~50 kΩ) in series with the sample. The resulting current I is of the order of 0.2 nA. The gate electrode is kept grounded for all measurements, with exception of Fig. S1 where some data with an applied gate voltage is presented.

4. Time dependent measurements collected at various applied gate voltages for a 160 nm wide device.

Fig. S1 shows measurements of longitudinal and Hall resistance as a function of time, collected at different gate voltages and a magnetic field of 2.3 T. The gate was tested prior to these measurements. The resistance between the Hall bar mesa and the gate electrode has a lower limit of 100 GΩ (limited by the resistance between the lines in the cryostat), well above any resistance measured on the sample when the telegraph noise is studied. Therefore, any potential influence from gate leakage can be excluded.

It is nonetheless clear that the gate voltage has some influence on the dynamics. While it seems unlikely that the gate voltage directly affects the domains, the coupling between the domains could be affected by changes in carrier density in the sample, which in turn depends on the gate voltage. A change in the coupling can obviously lead to more domains being active in the given magnetic field window, complicating the observed dynamics. A more detailed explanation will require further research.

5. Time dependent measurements collected at various applied magnetic fields for a 160 nm wide device.

Fig. S2 shows measurements of longitudinal and Hall resistance as a function of time, collected at various magnetic field values, at base temperature (40 mK). A "bin counter" next to each curve helps identify the distinct states. This data makes it clear that even a magnetic field as large as 8 T (Fig. S2A) is not sufficient to fully control (saturate) the magnetic moment in the entire sample, but rather that some domains remain active even at high fields.

6. Comparison between an estimated magnetic domain size in a 160 nm wide device and the surface topography of the film.

Fig. S3 shows a comparison of the relevant size scales related to the estimated magnetic domain size, crystal domain size, and the device size. The toot mean square roughness and peak-to-peak height difference extracted from the AFM scan in Fig. S3A are 0.46 nm and 4.5 nm, respectively. The film has a crystal rotational twinning domain structure, which is responsible for the granularity visible in Fig. S3A.

7. Thermal activation analysis for various values of parameter τ_0 in a 160 nm wide device.

Fig. S4 shows the thermal activation analysis (of the same data as in Fig. 2E of the main text) for different values of parameter τ_0. The high temperature regime can be reasonably well described for a range of values of τ_0 from roughly 1 ms to 20 ms (by readjusting the activation energies). The activation energies in Fig. S4B,D show a linear trend with magnetic field that is robust to within the uncertainty on τ_0.

8. Anti-symmetrization procedure for magnetic field sweeps of Hall resistance in a 160 nm wide device.

In order to analyze the possible reasons behind the deviation of Hall resistance from exact quantization in Fig. 1 of the main text, the measured Hall signal can be anti-symmetrized. Fig. S5 shows (and explains) the anti-symmetrization procedure for the Hall signal. It is clear that when the Hall measurement is anti-symmetrized, the value of Hall signal gets significantly closer to the expected quantized value of $\pm h/e^2$. This in turn indicates that the deviation from quantization observed on the raw data in Fig. 1 of the main text is primarily a result of longitudinal voltage admixing.

We emphasize that *only* Fig. S5 contains curves which were anti-symmetrized.

9. Two-terminal resistance of the 160 nm wide device at 40 mK.

Fig. S6 shows a two-terminal resistance measurement corresponding to the measurements plotted in Fig. 1A,B in the main text. Near the global magnetization reversal,

around the external magnetic field of 1 T, the measured signal increases to some 10 MΩ. This specific value is an artifact stemming from the fact that sample resistance there exceeds the limit placed by the capacitive coupling between the lines in the cryostat (that in turn leads to the AC current bypassing the sample, effectively rendering any precision measurements in that regime impossible). Over the entire magnetic field range (excluding this global magnetization reversal around 1 T), the resistance remains low enough that precision measurements can be easily performed.

10. Summary of the results from a series of Hall bar devices of various sizes, patterned from the same MBE layer as the 160 nm device.

In Fig. S7 we show a systematic study of the magneto-transport properties of the samples where the Hall bar size is varied. The studied Hall bars have widths of 160 nm, 240 nm, 1 µm, 2 µm, 8 µm, and 200 µm. All samples are patterned from the same MBE layer to ensure a systematic comparison.

11. Macroscopic quantum tunneling of magnetization in a 240 nm wide device.

Fig. S8 shows macroscopic quantum tunneling in a second sample, a Hall bar of 240 nm width. Both Hall resistance states (R_{yx}(+/-)) are tunable relative to each other in the same way as for the results in Fig. 2D in the main text. Increasing the external magnetic field leads to a R_{yx}(+) state being energetically more stable (longer lifetime) than the R_{yx}(-) state, promoting the ferromagnetic alignment of the domain relative to the majority background magnetization. The temperature dependence in Fig. S8B also reproduces the low temperature quantum limit (below some 290 mK) dominated by quantum tunneling of magnetization, and high temperature (thermally activated) limit dominated by thermal agitation.

We emphasize that the observed telegraph (two-level) noise dynamics reflect the dynamics of a *single* and specific magnetic domain, embedded in a background formed by other neighboring domains. Therefore, the resulting magnetic field scales must not be confused with parameters, such as the “coercive field”, which reflect the collective behavior of a large ensemble of domains in the material. Indeed, the “coercive field” appears to remain the same for the various samples in Fig. S7. At the same time, each studied tunneling domain (one in Fig. 2 of the main text from the 160 nm wide Hall bar, the other in Fig. S8 from the 240 nm wide Hall bar) shows telegraph noise in different magnetic field ranges (one around 2.1 T, the other around 3.2 T). This is expected as it is statistically implausible to randomly select two identical domains (with identical magnetic surroundings), and even small changes in the domain size or shape (or the domain surroundings) can have profound effect on the relevant energy scales and dynamics. The overall phenomenology associated with the quantum tunneling is nevertheless fully reproducible, showing that the effect is robust to sample-to-sample variations.

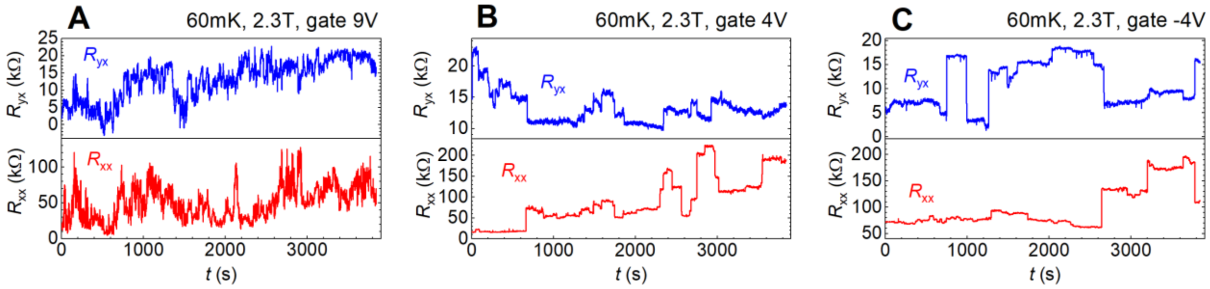

Fig. S1. Time dependent measurements collected at various applied gate voltages for a 160 nm wide device.

Time dependent measurements of Hall and longitudinal resistance collected with various applied gate voltages, at a constant magnetic field of 2.3 T and temperature 60 mK. The applied gate voltage is: (A) 9V, (B) 4V, and (C) -4V.

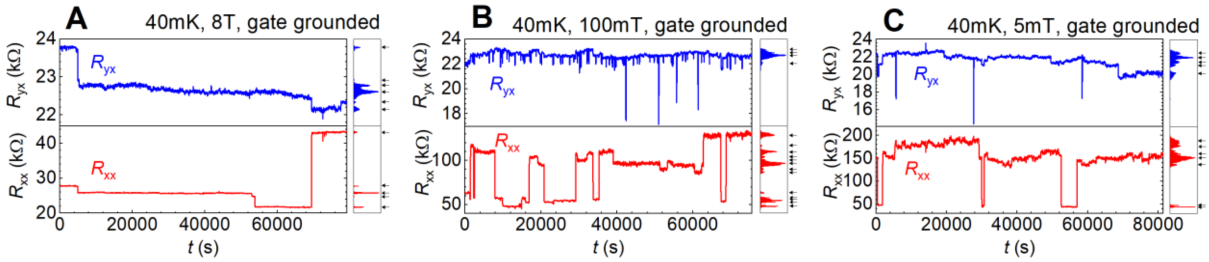

Fig. S2. Time dependent measurements collected at various applied magnetic fields for a 160 nm wide device.

Time dependent measurements of Hall and longitudinal resistance collected with a grounded gate, at various magnetic field values, and a constant temperature of 40 mK. The external magnetic fields are: (A) 8 T, (B) 100 mT, and (C) 5 mT. A "bin counter" is plotted next to each figure, to identify the number of distinct states. The largest number of distinct states, 13, is visible in (B) R_{xx}.

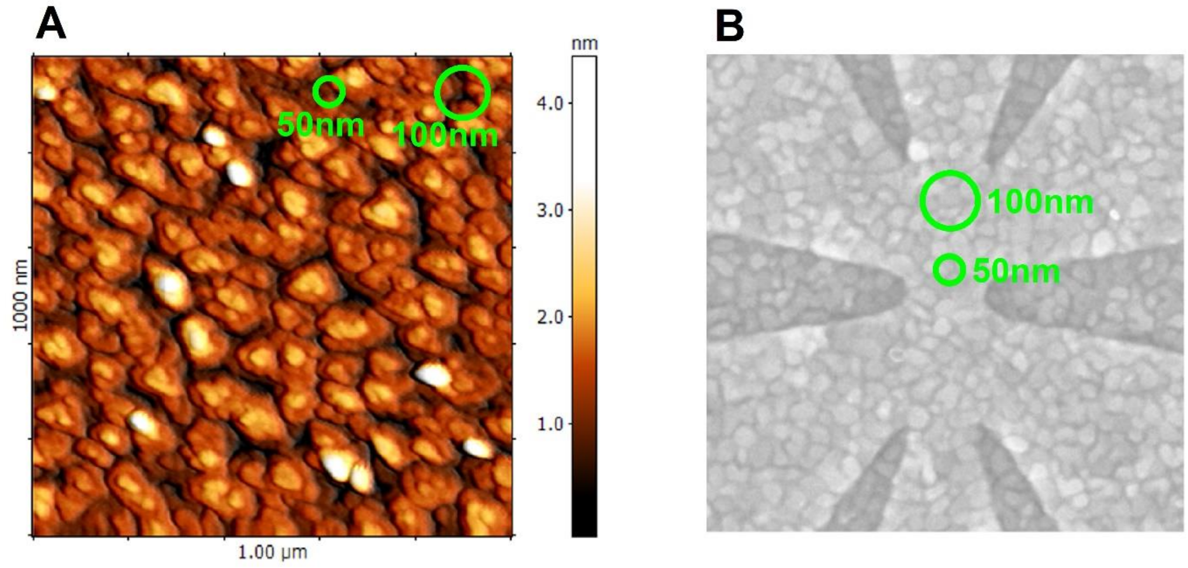

Fig. S3. Comparison between an estimated magnetic domain size in a 160 nm wide device and the surface topography of the film.

(A) Atomic force microscope (AFM) image of the surface topography of a reference $(V,Bi,Sb)_2Te_3$ layer grown under the same conditions (but without the protective Te cap) as the layer from which the investigated nanostructure was patterned. (B) Scanning electron microscope (SEM) image of a reference device patterned using the same lithographic process as the device investigated in the Article. Both images are 1 µm by 1 µm. The green circles (the positioning of which is arbitrary in the figure) are merely visual aids representing the estimated size range for the magnetic domain size of diameter 50-100 nm. The granularity visible in picture (B) is from the surface of the gate metal.

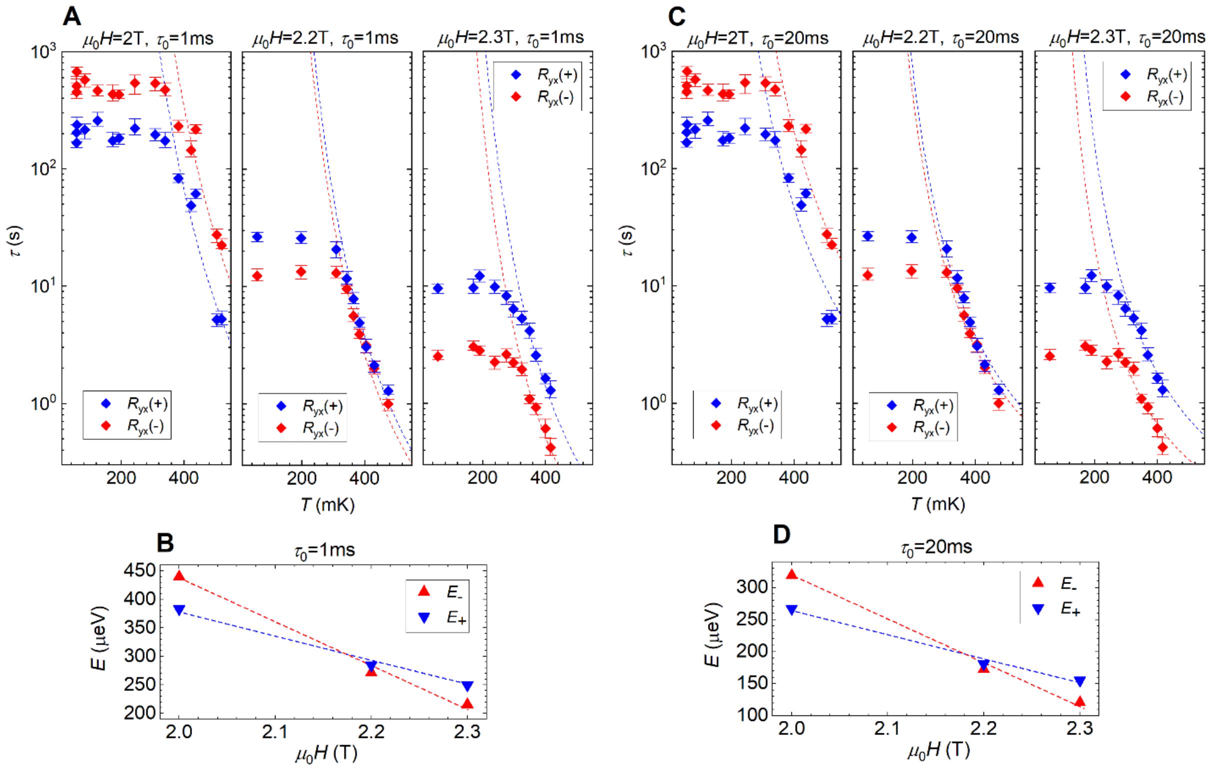

Fig. S4. Thermal activation analysis for various values of parameter τ_0 in a 160 nm wide device.

(A) Evolution of τ with temperature for three magnetic field values with high temperature activation dashed lines for τ_0=1 ms, and (B) corresponding thermal activation energies E_+ and E_-. (C) Evolution of τ with temperature for three magnetic field values with high temperature activation dashed lines for τ_0=20 ms, and (D) corresponding thermal activation energies E_+ and E_-. The dashed lines in (A) and (C) follow $\tau = \tau_0\exp[E_{+/-}/(k_BT)]$. The dashed lines in (B) and (D) are guide to the eyes.

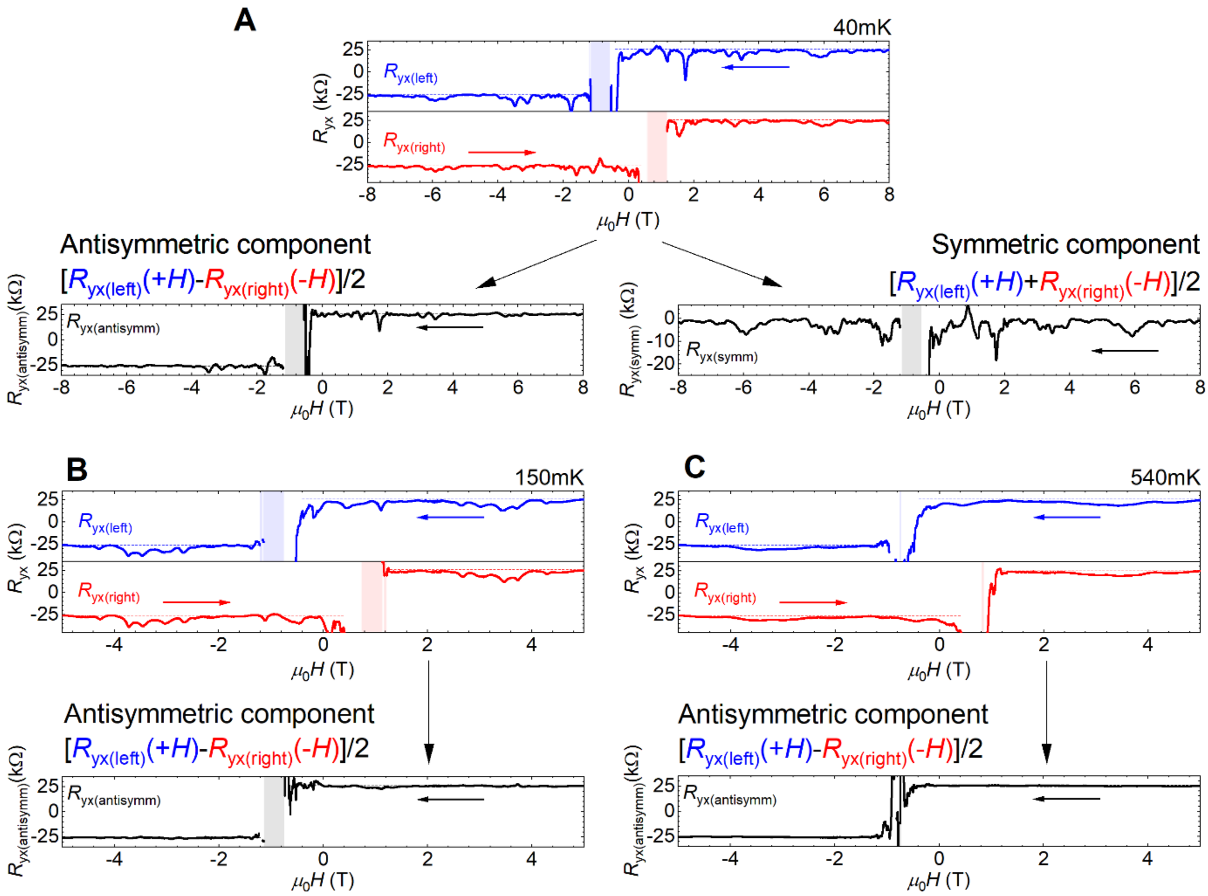

Fig. S5. Antisymmetrization procedure for magnetic field sweeps of Hall resistance in a 160 nm wide device.

(A) The top panel repeats the R_{yx} data from Fig. 1A in the main text (collected at 40 mK), which in the bottom part of the figure is split into antisymmetric and symmetric components with respect to the hysteretic *B* field. In order to account for hysteresis, the components are extracted by calculating the average (or difference) between the sweep towards the left for a given *H* field, with the sweep to the right for the negative of that field. (B) The same for the antisymmetric component for the R_{yx} data collected at 150 mK (reproduced from Fig. 1C in the main text), and (C) for the R_{yx} data collected at 540 mK (reproduced from Fig. 1D in the main text). The arrows in the figures indicate the sweep direction. The shaded regions represent a regime around the global magnetization reversal where the sample is insulating. The horizontal dashed lines represent resistance values expected for a perfect quantum anomalous Hall effect.

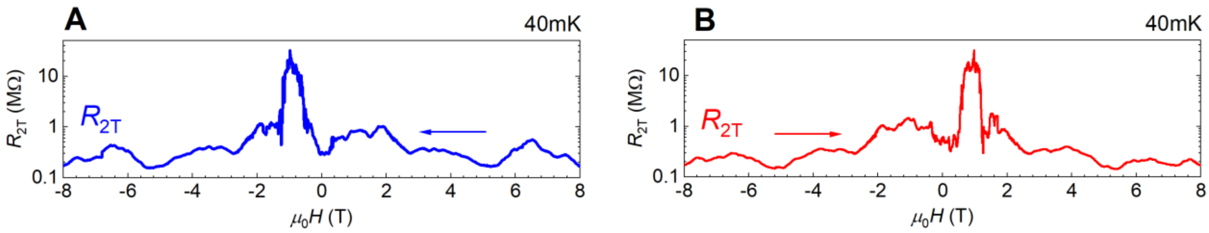

Fig. S6. Two-terminal resistance of the 160 nm wide device at 40 mK.
Magnetic field sweep of the two-terminal resistance collected at temperature 40 mK, corresponding to the Hall and longitudinal resistance data plotted in Fig. 1A and B in the main text. The arrows indicate the sweep direction.

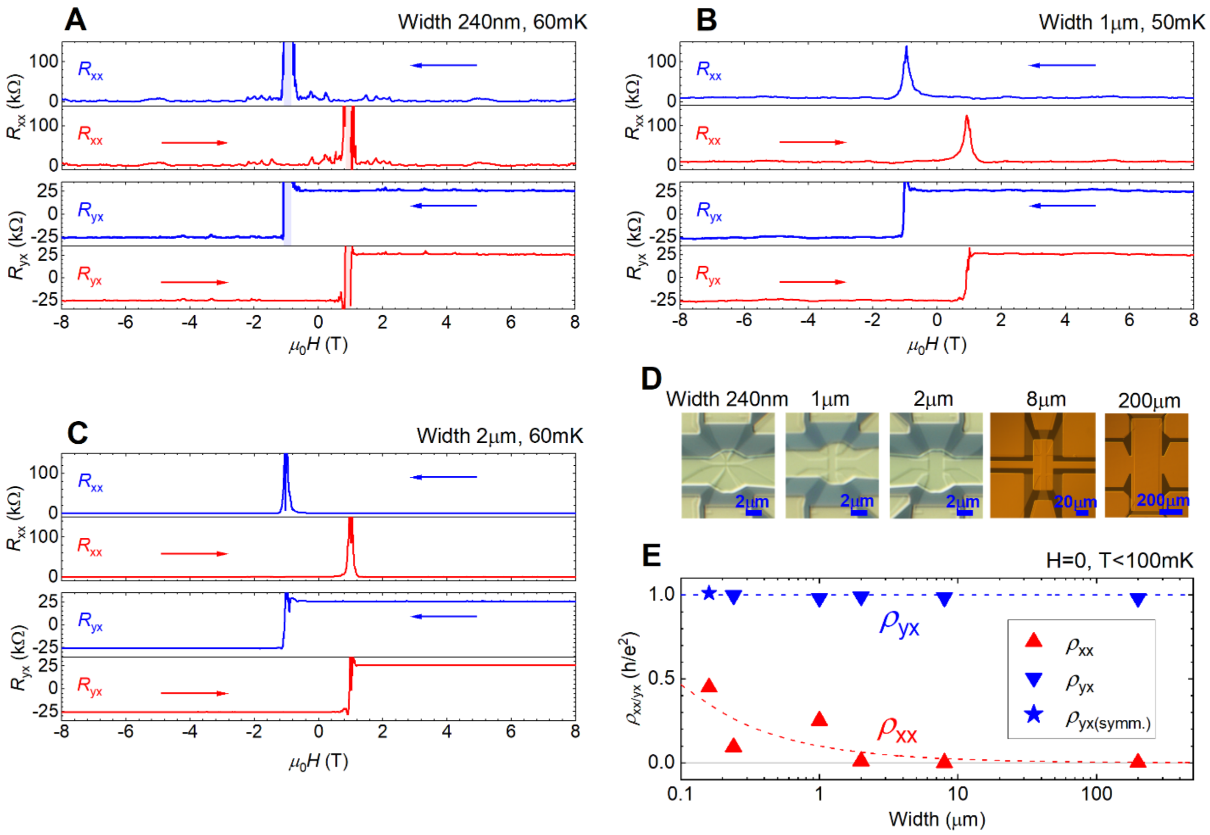

Fig. S7. Summary of the results from a series of Hall bar devices of various sizes, patterned from the same MBE layer as the 160 nm device.
(A) Magnetic field sweep of longitudinal and Hall resistances collected at base temperature from a device of 240 nm width. The colored arrows indicate the sweep direction. (B) The same for a 1 μm wide device, and (C) for a 2 μm wide device. (D) Optical microscope images of a series of devices of various sizes (images of a 160 nm wide device can be found in Fig. 1 of the main text). (E) Base temperature zero magnetic field device width dependence of Hall and longitudinal resistivities, obtained from the series of devices, all from the same MBE layer. Dashed lines are guides to the eye. The single data point labeled as a star is Hall resistivity for a 160 nm device, where the value was anti-symmetrized (see Fig. S5), for a direct comparison with the value obtained in Ref. (*35*) in a nanostructure of similar size.

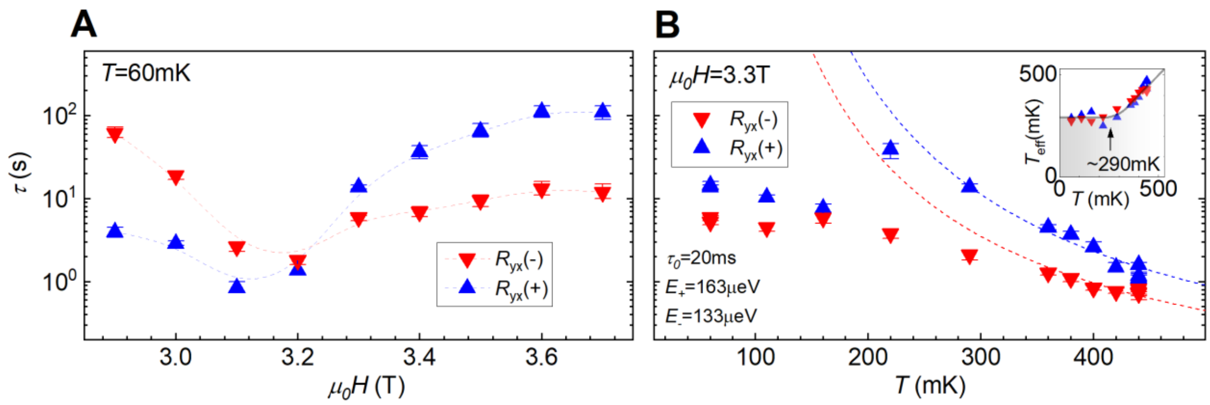

Fig. S8. Macroscopic quantum tunneling of magnetization in a 240 nm wide device. (A) Base temperature (60 mK) magnetic field dependence of lifetimes τ obtained from telegraph noise observed in the 240 nm wide Hall bar. The lifetimes correspond to the state with larger (R_{yx}(+)) and smaller (R_{yx}(-)) Hall resistance value. The behavior is consistent with observations from the 160 nm device. An increase of magnetic field leads to an increase of τ of the R_{yx}(+) state with respect to that of a R_{yx}(-) state, favoring the ferromagnetic alignment of the domain with respect to the background magnetization. Dashed lines are guides to the eye. (B) Evolution of τ with temperature at magnetic field of 3.3 T. The inset shows the effective temperature (T_{eff}). A clear transition is observed between the thermally activated regime (above about 290 mK) dominated by thermal agitation, and a quantum regime (below about 290 mK) dominated by macroscopic quantum tunneling, where the thermal activation model breakdown is observed. This reproduces the behavior observed in the 160 nm wide device. The dashed lines show the thermal activation $\tau = \tau_0 \exp[E_{+/-}/(k_B T)]$, with the parameter values given in the figure.
